# The analysis and identification of charred suspected tea remains unearthed from Warring State Period Tomb

**DOI:** 10.1038/s41598-021-95393-w

**Published:** 2021-08-16

**Authors:** Jianrong Jiang, Guoquan Lu, Qing Wang, Shuya Wei

**Affiliations:** 1 Institute of Cultural Heritage and History of Science & Technology, University of Science and Technology Beijing, Beijing, 100083 China; 2 School of History and Culture, Shandong University, Jinan, 250100 China

**Keywords:** Organic chemistry, Materials science

## Abstract

Recently, a bowl containing charred suspected tea remains unearthed from the early stage of Warring States period tomb in Zoucheng City, Shandong Province, China. To identify the remains is significant for understanding the origin of tea and tea drinking culture. Scientific investigations of the remains were carried out by using calcium phytoliths analysis, Fourier transform infrared spectroscopy (FTIR), Gas Chromatograph Mass Spectrometer (GC/MS) and Thermally assisted hydrolysis—methylation Pyrolysis Gas Chromatography Mass Spectrometry (THM-Py-GC/MS) techniques. Modern tea and modern tea residue were used as reference samples. Through phytoliths analyses, calcium phytoliths identifiable from tea were determined in the archeological remains. The infrared spectra of the archaeological remains was found similar as modern tea residue reference sample. In addition, the biomarker compound of tea—caffeine was determined in the archaeological remains by THM-Py-GC/MS analysis. Furthermore, through GC/MS analysis, some compounds were found both in the archeological remains and the modern tea residue reference samples. Putting the information together, it can be concluded that the archaeological remains in the bowl are tea residue after boiling or brewing by the ancient.

## Introduction

China is the first country in the world to discover and cultivate tea. In Chinese legend, tea was first discovered as an antidote by Emperor Shen Nung in 2737 bc, according to the first monograph on Chinese herbal medicine Shennong’s Classic of Materia Medica (*神农本草经*)^1^. The first mention of tea planting is believed to occur in the *Xiaxiaozheng* (*夏小正*), a Chinese earliest almanac recording traditional agricultural affairs, probably written in the Warring States Period (475–221 bc). According to the literature, in the Spring and Autumn Period (770–476 bc), tea had been used as a sacrifice and vegetable, in the Warring States period and the early Western Han Dynasty, tea cultivation, tea making techniques and tea drinking custom in Sichuan province began to spread to other places^2^.

**Figure 1:**
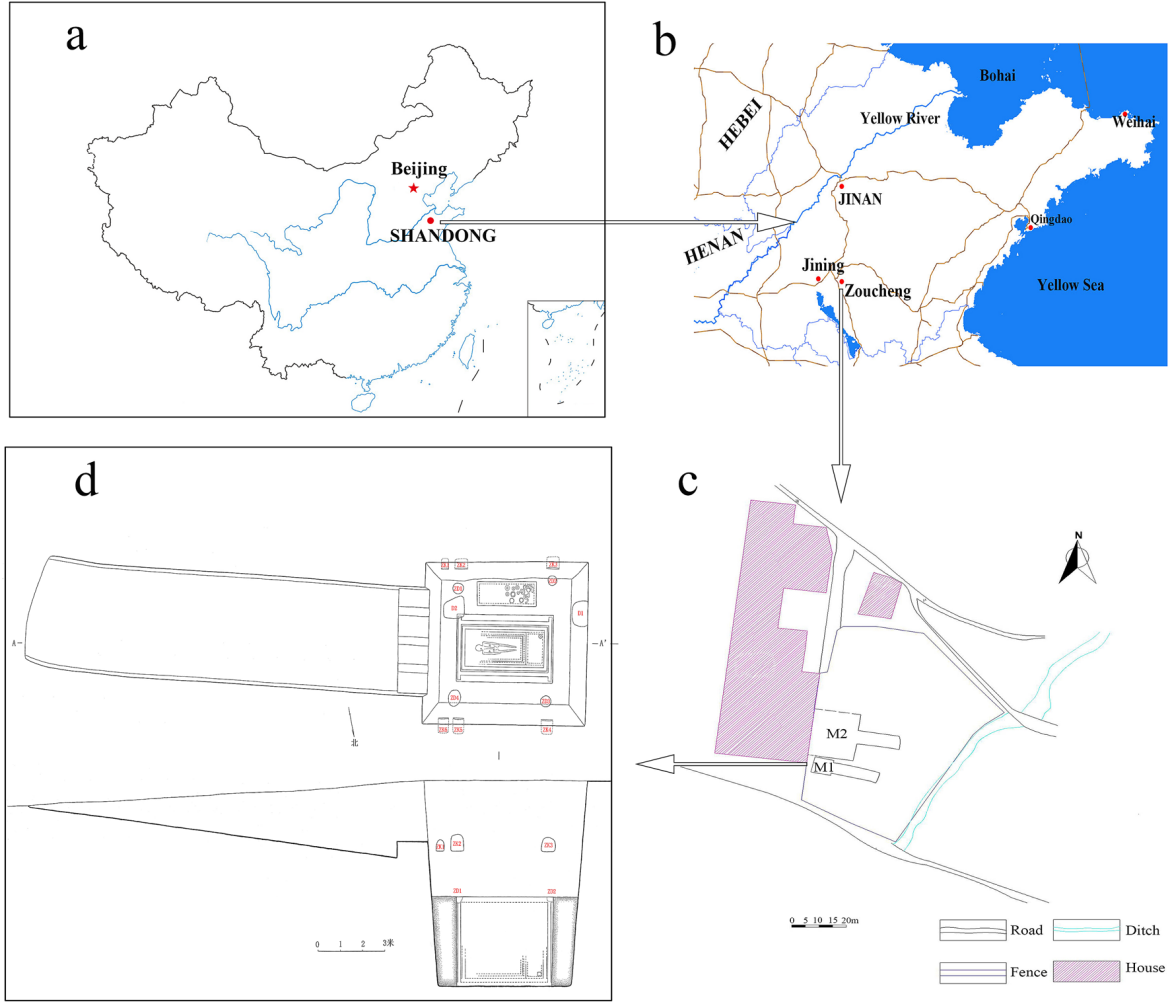
The map shows (**a**) Location of Shandong Province in China; (**b**) The Ancient Capital City Site of the Zhu Kingdom in Zoucheng City; (**c**) The plan of the tomb; (**d**) The plan and profile of tomb No. 1 at Xigang.

The physical evidence of tea is very important to confirm the origin, development, function and culture of tea. As archaeological plant leaves remains have been buried for many years, most of them have rotted or charred, it is difficult to find archaeological plant leaves remains in archeological excavation. The first tea remains were found in Northern Song tomb of Lu’an, Anhui Province^3^. The oldest physical evidence of tea remains are from other two funerary sites: the Han Yangling Mausoleum in Xi’an, Sha’anxi Province, and the Gurgyam Cemetery in Ngari district, western Tibet, revealing that tea was used by Han Dynasty emperors as early as 2100 year BP and had been introduced into the Tibetan Plateau by 1800 year BP^4^, but whether the tea was used as beverage, food, medicine is unclear. Recently, some charred suspected tea remains (CST) were found in a bowl unearthed from tomb No. 1 at Xigang in the Ancient Capital City Site of the Zhu Kingdom in Zoucheng City (The early stage of Warring States, approximately 2400 years ago), Shandong Province (Fig. 1)^5^. If the remains could be determined as tea, that would be the direct evidence for tea drinking in the ancient time.

Previously, the researchers commonly used for the identification of plant remains were mainly based on the morphology of the plant, however, most of archaeological plant remains have rotted or charred due to the interference of various microorganisms, oxidation and other factors in the buried environment for many years, the morphology of the plants also changed dramatically, therefore, to identify the plant species by morphology is not applicable to the sample CST. Recently calcium phytoliths (calcium oxalate plant crystals), biomarkers (caffeine and theanine) were identified in archaeological tea remains by using Gas Chromatography/Mass spectrometry (GC/MS) and Ultra-performance Lquid Chromatography and Mass Spectrometry (UPLC–MS) techniques^4^. For modern tea study, Fourier transform infrared spectroscopy (FTIR)^6–9^, Spectrophotometry, Thermospray–LC–MS^10^, Head space solids-phase microextraction in combination with gas chromatography–mass spectrometry (HS-SPME/GC–MS)^11^, Gas chromatography–mass spectrometry (GC–MS)^12,13^ are the mainly techniques applied.

In this study, methods of calcium phytoliths analysis, Fourier transform infrared spectroscopy (FTIR), Gas Chromatography/Mass spectrometry (GC/MS) and thermally assisted hydrolysis–methylation Pyrolysis Gas Chromatography/Mass Spectrometry (THM–Py–GC/MS) were chosen for the identification of the sample CST found in the Warring State tomb. In the meantime, modern reference samples were studied by using the same analytical methods as the archaeological sample for comparison.

## Materials and methods

**Figure 2:**
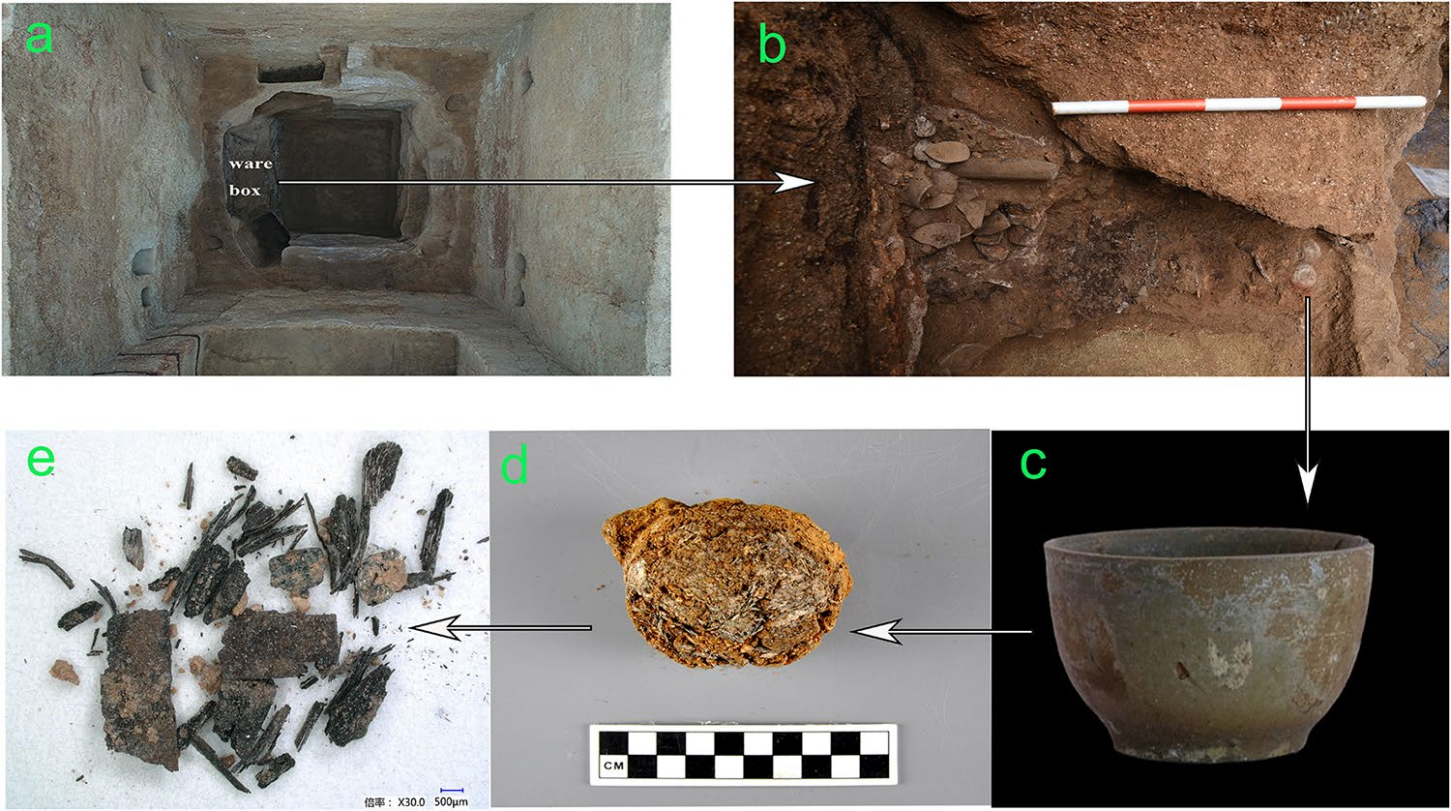
The map shows (**a**) Tomb No. 1 at Xigang; (**b**) Burial objects in the ware box; (**c**) The unearthed bowl; (**d**) The residues which poured out from the bowl; (**e**) The sample CST take from the residues.

Archaeological sample: the Archaeological sample CST take from the residues which poured out from the bowl unearthed from the Tomb No.1 at Xigang (Fig. 2).

Reference samples: since the sample CST was unearthed in a bowl, therefore, it is not excluded that the sample is tea residue left after boiling or brewing by the ancient, so in this study, modern tea and modern tea residue were used as reference samples. Modern tea residue was prepared from brewing tea with water for several times, then dry thoroughly and finally grinded into powders. The modern tea samples were bought from tea shop in Beijing, which are black tea produced from Shangdong Laoshan.

### Analysis of calcium phytoliths

Calcium phytoliths experiment was performed according to the procedure described in the literature^4^. Identification of calcium phytoliths was performed under a LEICA DM2700P microscope.

### Fourier transform infrared spectroscopy (FTIR)

For FTIR analysis, Nicolet 6700 Advanced Fourier transform infrared spectrometer (America Thermo Fisher Scientific) was used. The spectra were collected over the 4000–500 cm^−1^ region, using attenuated total reflectance (ATR) for the measurements, the spectral resolution is 4 cm^−1^ and the number of scans is 64. Each sample was scanned at 25℃ and the data acquisition system used was OMNIC.

### Gas chromatograph–mass spectrometer (GC/MS)

GC–MS analysis was performed using Agilent GC–MS-QP2010Ultra (Shimadzu, Japan). A capillary column Ultra-5MS (5% diphenyl/95% dimethyl siloxane), 0.25 mm internal diameter, 0.25 μm film thickness and 30 m length [Frontier lab, Japan] was used for the separation. Temperature programmed: initially keeping the column at 120 °C for 2 min, followed by a gradient of 5 °C/min to 270 °C and hold for 10 min. The injector temperature was set to 240 °C. 30:1 split ratio. The carrier gas used was Helium (purity 99.999%). The electronic pressure control was set to a constant flow of 1 ml/min; Electron ionization (EI) temperature was at 280 °C; Transmission lines temperature was at 220 °C; Range of Scanning: 35 ~ 510 m/z.

Analysis procedure: A sample (20 mg) was weighed, transferred into a sampling vial with ultrapure water, boiled the sample for 10 min in a water bath, extracted under sonication at 60 °C for 30 min and then centrifuged for 10 min. Afterwards, the supernatant was transferred to a sample vial, then it was evaporated and dried in a stream of N_2_ at 60 °C. Finally, the dried tea extract was dissolved in solvent acetonitrile (ACN) and derivatized with *N*-(tert-Butyldimethylsilyl)-*N*-methyltrifluoroacetamide (MTBSTFA, 1: 1 to ACN, v/v) at 110 °C for 30 min, transfer the mixed fluid into an auto sampling vial for GC/MS analysis.

### Pyrolysis gas chromatography mass spectrometry (Py-GC/MS)

For Py-GC/MS analysis, a Multi-Shot pyrolyzer, type EGA/PY-3030D, made by Frontier Lab, Japan, and a gas chromatograph mass spectrometer, GC–MS-QP2010 Ultra (Shimadzu, Japan). Shimadzu GC–MS real time analysis software was used for GC–MS control, peak integration and mass spectra evaluation.

The pyrolysis was performed at 550 °C for 12 s. The pyrolyser interface was set to 290 °C and the injector was set to 250 °C. A capillary column SLB-5MS (5% diphenyl /95% dimethyl siloxane), 0.25 mm internal diameter, 0.25 μm film thickness and 30 m length [Supelco] was used in order to provide an adequate separation of the components. The chromatographic conditions were as follows: the oven initial temperature was set to 35 °C for 5 min, followed by a gradient of 60 °C/min to 100 °C, for 3 min, 14 °C/min to 240 °C , then 6 °C/min to 315 °C and hold for 1.5 min, the carrier gas was Helium (He, purity 99.999%). The electronic pressure control was set to a constant flow of 0.92 ml/min, in split mode at 1:20 ratios. Ions were generated by electron ionization (145.3 eV) in the ionization chamber of the mass spectrometer. The mass spectrometer was set from m/z 35 to 750. EI mass spectra were acquired by total ion monitoring mode. The temperatures of the interface and the source were 280 °C and 200 °C, respectively.

NIST14 and NIST14s Library of Mass Spectra were used for identifying the compounds.

#### Analysis procedure

About 50 μg sample was placed in a sample cup, 3 μL of 25% aqueous TMAH (analytical pure, Sinopharm Chemical Reagent Co., Ltd) solution were injected into the sample cup, the cup was placed on top of the pyrolyzer at ambient temperature and then pyrolyzed immediately, afterwards the temperature program for the GC/MS analysis was started. During the process of analysis, blank tests were conducted before each sample.

### Statement

The use of plants parts in the present study complies with National standards of the People's Republic of China on black tea (GB/T13738).

## Results and discussion

### Analysis of calcium phytoliths

**Figure 3:**
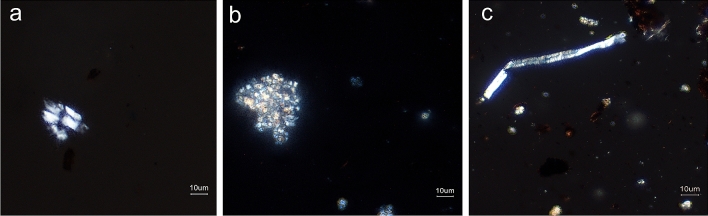
Photographs under microscope of calcium phytoliths from sample CST. (**a**) crack-type calcium phytoliths; (**b**) druse-type calcium phytoliths; (**c**) trichome-type calcium phytoliths.

Calcium phytolith analyses were carried out according to the procedure described in the literature^4^. The morphology observation of sample CST under microscope is depicted in Fig. 3, which reveals that the sample contains abundant calcium phytoliths, including the crack, druses and trichome base, these calcium phytoliths also match the *genus Camellia*, especially druse and trichome base are the most distinctive crystals in tea plants^4^.

### FTIR analysis

**Figure 4:**
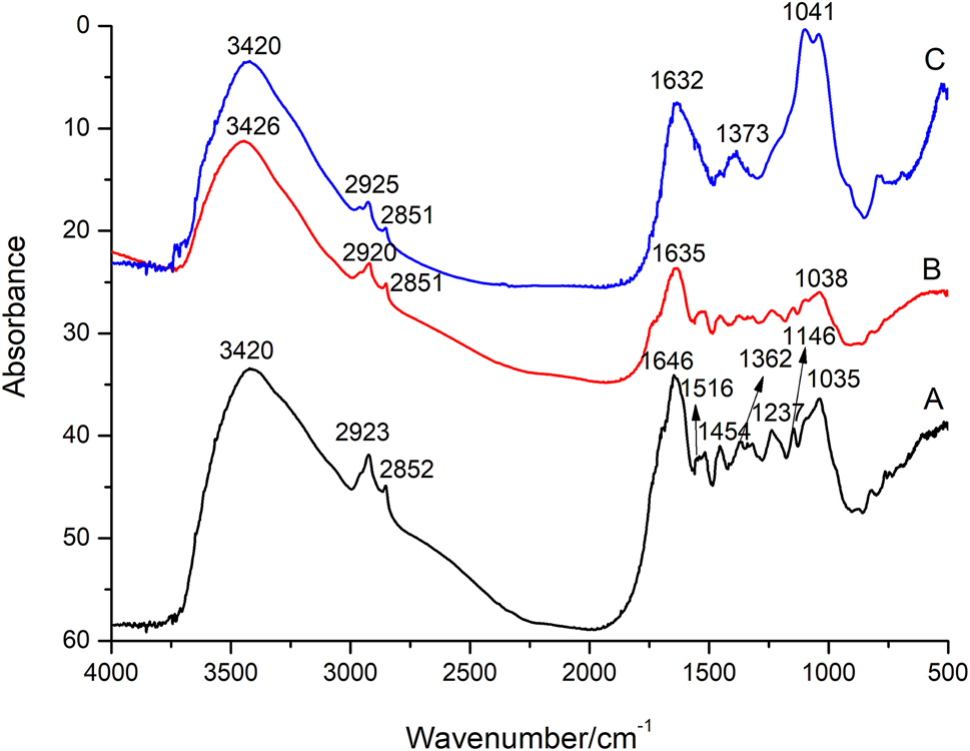
Infrared spectra of A—modern tea; B—modern tea residue; C—sample CST.

The infrared spectra of modern tea, modern tea residue and the sample CST are shown in Fig. 4. The vibrations of the functional groups of the compounds in tea and their corresponding infrared absorption characteristic peaks are consistent with the literatures^2,6–8^. Taking infrared spectrum of modern tea as an example, the band assignment to chemical bonds for the vibrational FTIR spectra of modern tea is summarized in Table 1.

**Table 1:** Band assignments for the FTIR spectra obtained from modern tea.

Wavenumber (cm^−1^)	Vibrational mode assignment	Absorption peak intensity: s (strong)/m (medium)/w (weak)
3420	–OH stretching vibration of tea-polyphenols and tea-polysaccharides	s
2923, 2852	Saturated C–H stretching vibration	m, w
1646	C=C stretching vibration peak of sugars and flavonoids	s
1516	–NO_2_ stretching vibration peak of aromatic compounds in tea	w
1454	saturated C–H deformation vibration	w
1362	–NO_2_ stretching vibration peaks of aliphatic compounds	w
1237	C–O stretching vibration peak in amides	w
1146	C–O–C antisymmetric stretching vibration	w
1035	O–H in-plane deformation vibration	s

The peak shapes and the positions of main absorption peaks (1632, 1041 cm^−1^) of the archaeological sample CST are consistent with the modern tea reference samples (Fig. 4), so it is speculated that the sample CST is most likely ancient tea.

The intensity of some absorption peaks in modern tea residue decreased significantly in comparison with modern tea, such as the peaks at 1516, 1454, 1237 cm^−1^, indicating some compounds in the tea may be dissolved in water. Sample CST has been buried for hundreds of years, microorganisms and other factors in the burial environment may cause chemical changes, resulting in the subtle differences in infrared spectra between the archaeological sample CST and reference samples. To confirm whether the sample CST is ancient tea or not, further study by other techniques were carried out as following.

### Biomarker analysis

#### THM-Py-GC/MS analysis

**Figure 5:**
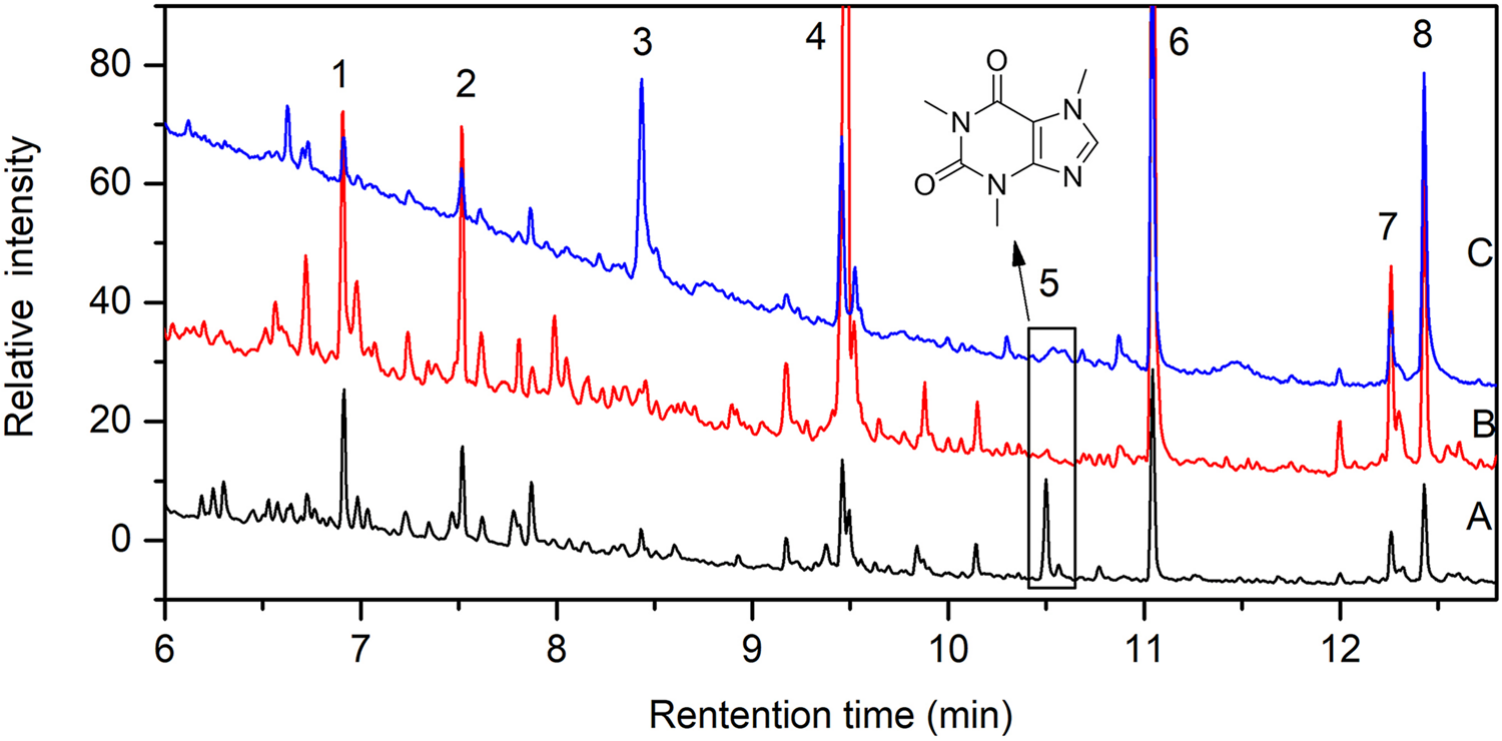
TIC chromatogram obtained by THM-Py-GC/MS of A—modern tea; B—modern tea residue; C—sample CST; The peak numbers are corresponding to: (1): 1,3,5-trimethoxy-benzene; (2): 2,4,6-trimethoxytoluene; (3): 3,4-dimethoxy-benzoic acid, methyl ester; (4): 3,4,5-trimethoxy-benzoic acid, methyl ester; (5): caffeine; (6): hexadecanoic acid, methyl ester; (7): 9-octadecenoic acid, methyl ester; (8): methyl stearate.

The chromatograms of modern tea, modern tea residue and the sample CST obtained by THM-Py-GC/MS are shown in Fig. 5. Three parallel analyses were conducted for each sample, the analysis results are consistent with each other.

**Figure 6:**
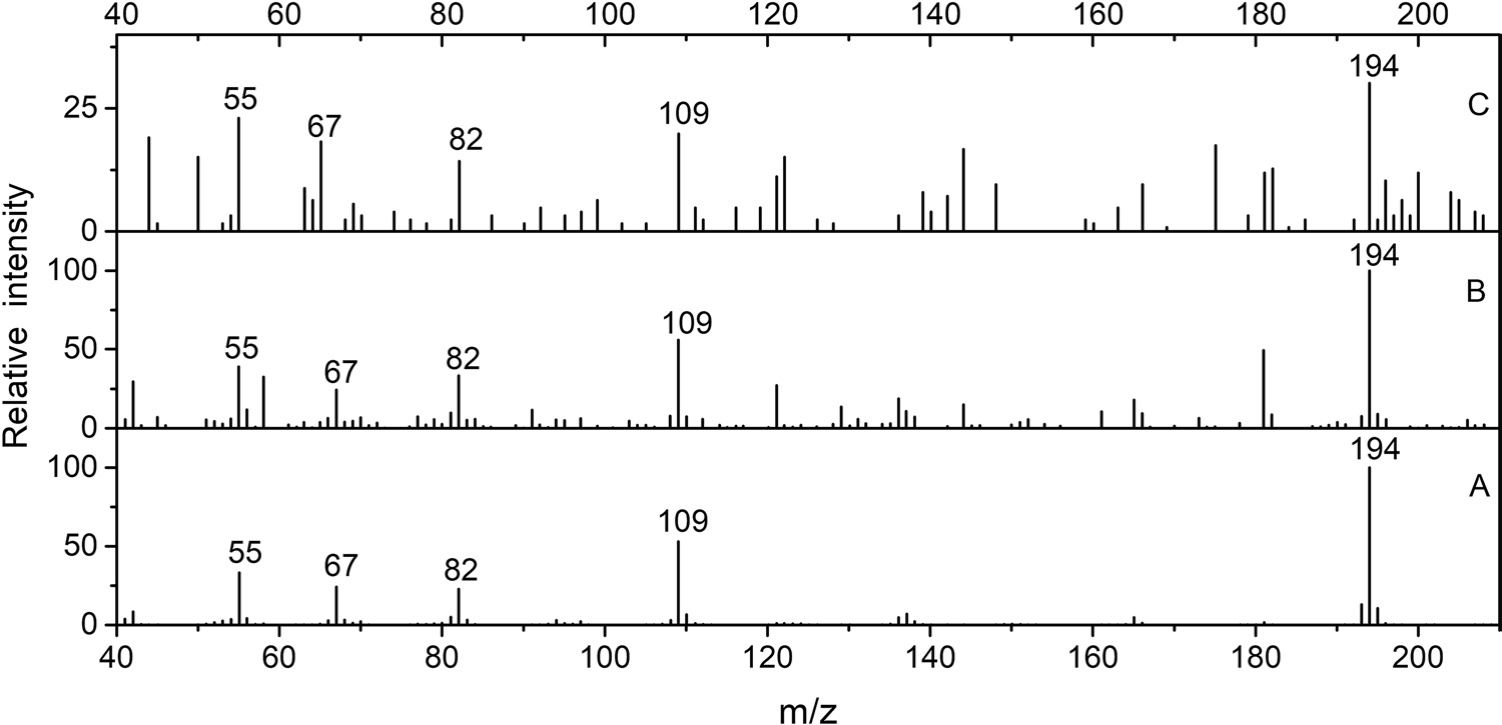
The mass spectrum of caffeine (peak 5 in Fig. 5) of A—modern tea; B—modern tea residue; C—sample CST by THM Py-GC/MS analysis.

Comparing retention time and mass spectrum of the main chromatographic peaks in the sample CST and the reference samples (modern tea and modern tea residue), the main peaks found in the sample CST are also present in the reference samples (peak No. 1–8), which are 1,3,5-trimethoxy-benzene, 2,4,6-trimethoxytoluene, 3,4-dimethoxy-benzoic acid methyl ester, 3,4,5-trimethoxy-benzoic acid methyl ester, caffeine, hexadecanoic acid methyl ester, 9-octadecenoic acid methyl ester, methyl stearate, the peak 1, 2, 3, 4 belong to methoxybenzene compounds which are the characteristic components of tea aroma^14,15^, and hexadecanoic acid methyl ester, 9-octadecenoic acid and methyl stearate are common fatty acids in tea^16^. Especially the biomarker compound of tea—caffeine was identified (peak No. 5, RT 10.5 min) in CST sample, the mass spectra are shown in Fig. 6. Caffeine is easily soluble in water, most of the caffeine in tea was leached out in the process of brewing tea, therefore, the content of caffeine in modern tea residue is significantly lower than that in modern tea (Fig. 5). Peak 3 is one of the most intense for CST but not significant in the modern sample, probaly due to two reasons: firstly, the relative contents of the chemical components in different tea are different; secondly, it is probably due to the effect of the burial environment. In order to see the influence of the burial environment, soil samples from the area where the bowl was found were analyzed by Py-GC/MS, small amount of fatty acids were found, which will not affect the conclusion for sample CST.

#### GC/MS analysis

**Figure 7:**
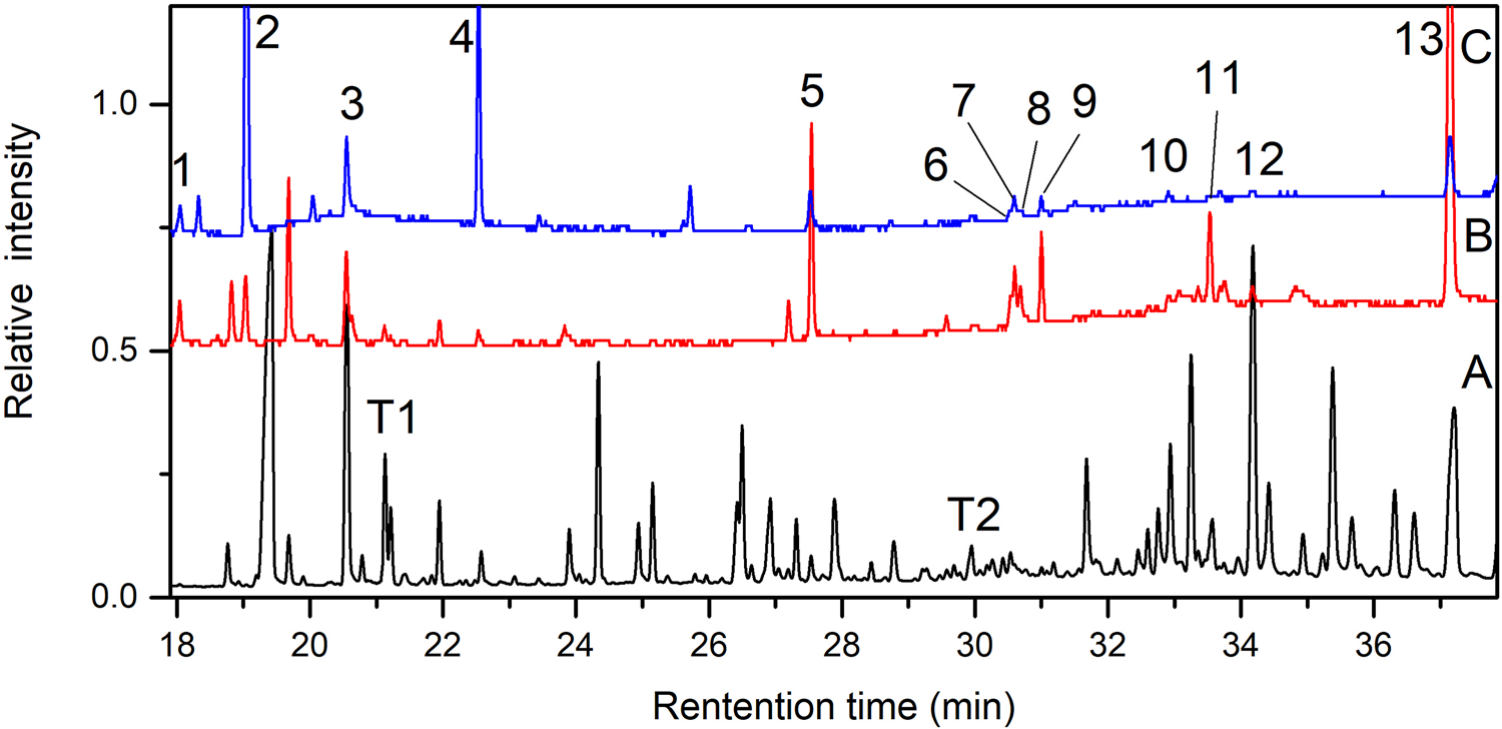
TIC chromatogram obtained by GC/MS of A—modern tea; B—modern tea residue; C—sample CST; T1, T2: two derivatized peaks of theanine in modern tea sample (A); The peak numbers are corresponding to the numbers in Table 2.

The modern tea reference sample, modern tea residue reference sample and the sample CST were pretreated according to the procedure described in a previous section in this document. The chromatograms of them obtained by GC/MS analyses are shown in Fig. 7. Three parallel analyses were conducted for each sample, the analysis results are consistent with each other.

The main amino acids contained in tea were detected in modern tea sample after derivatized by MTBSTFA, which is consistent with the literature^17,18^. Especially the tea marker compound-theanine, two derivatized peaks of theanine were detected in modern tea reference sample (labeled as T1 and T2 in Fig. 7). In the modern tea residue sample, only a trace of theanine (T1) was found, but not found in the CST sample. Theanine is the main free amino acid in tea, its concentration is significantly decreased after tea was brewed due to its good solubility in water, therefore, the content of theanine in modern tea residue and sample CST is so low that even cannot be detected. However, there are some other compounds were detected both in modern tea residue and sample CST, which are listed in Table 2, most of these components are organic acids, which are the common components in tea, and as a water-soluble substance, which can be also leached out in the process of brewing tea^19^. Although some of the compounds were not identified what they were, but they were both present in modern tea residue and sample CST (peak No. 1, 4, 10, 12, 13 in Fig. 7), indicating those compounds origin from tea, which provide additional evidence that the archaeological sample CST is most likely tea residue left after boiling or brewing. The relative intense of peak 4 is higher in CST sample in comparison with the reference samples, probably due to ageing.

**Table 2:** GC/MS analysis result of modern tea residue and sample CST.

Peak no.	RT (min)	Main ions (m/z)	Compounds identified
1	18.04	115, 147, 173	Unidentified
		259, 386	
2	19.04	147, 189, 221	Boric acid, 3TMS derivative
		263, 355	
3	20.54	211, 269, 383, 425	Phosphoric acid, tris(tert-butyldimethylsilyl) ester
4	22.56	147, 221, 263, 337	Unidentified
5	27.53	117, 131, 313	Palmitic acid, TBDMS derivative
6	30.54	129, 337	Linoelaidic acid, tert.-butyldimerthylsilyl ester
7	30.59	129, 339, 381	Petroselinic acid, TBDMS derivative
8	30.68	129, 339	.alpha.-Linolenic acid, TBDMS derivative
9	31	117, 129, 341	Stearic acid, TBDMS derivative
10	32.92	117, 143, 237	Unidentified
		252, 359	
11	33.54	223, 339, 455, 469	2-Amino[1,3]thiazolo[4,5-d]pyrimidine-5,7-diol
12	34.15	185, 241, 256, 359	Unidentified
13	37.14	238, 323, 397	Unidentified
		439, 495	

## Conclusions

In this study, Calcium phytoliths analysis, Fourier transform infrared spectroscopy (FTIR), Gas Chromatograph Mass Spectrometer (GC/MS) and Thermally assisted hydrolysis-methylation pyrolysis-gas chromatography/mass spectrometry (THM–Py-GC/MS) techniques were applied for the identification of archaeological remains–charred suspected tea (CST) excavated from the early stage of Warring State Period tomb in Shandong Province. The experimental results show that the sample CST contains abundant calcium phytoliths identifiable as tea, The FTIR spectra of CST sample are similar with that of the modern tea residue. Moreover, caffeine, methoxybenzene compounds, organic acids, 2-Amino[1,3]thiazolo[4,5-d]pyrimidine-5,7-diol and several unidentified compounds were detected in both the sample CST and the reference sample (modern tea residue) by THM–PY-GC/MS and GC/MS. By putting the information together, it can be concluded that the archaeological remains in the bowl are tea residue after boiling or brewing by the ancient.

Tea drinking is one of the most representative traditional cultures in China, since ancient times, the Chinese people have always had the habit of drinking tea, but there is no physical evidence to prove when tea actually appeared, until the discovery of tea in the Han Yangling Mausoleum, which proved that Chinese tea has a history of at least 2150 years, which has earned recognition from Guinness World Records as the oldest tea in 2016^4^. The identification of the tea remains at the Ancient Capital Site of the Zhu Kingdom in Zoucheng (the early stage of Warring States, approximately 2400 years ago) has advanced the origin of tea by nearly 300 years. Furthermore, the tea was found in a small bowl, providing additional evidence of the usage of tea. The results of this study indicate that tea drinking culture may start as early as in Warring State period.
